# Untargeted metabolomics reveals quinic acid as the kiwifruit component that affects brain activity in mice

**DOI:** 10.1371/journal.pone.0326134

**Published:** 2025-08-18

**Authors:** Claudio Marcello Marzo, Martino Bianconi, Mauro Commisso, Sofia Gambini, Cristiano Chiamulera, Linda Avesani, Stefano Negri, Flavia Guzzo

**Affiliations:** 1 Department of Biotechnology, University of Verona, Strada Le Grazie, Verona, Italy; 2 National Biodiversity Future Center (NBFC), Piazza Marina, Palermo, Italy; 3 Department of Diagnostic and Public Health, University of Verona, Piazzale Scuro, Verona, Italy; University of California Riverside, UNITED STATES OF AMERICA

## Abstract

Plant-based diets are associated with both physical health and psychological wellbeing. Recent evidence suggests that kiwifruit positively affects cognitive functions and mood, but the bioactive components responsible for this are unknown. In this work, we combined two predictive preclinical models of depression (TST and FST) with untargeted metabolomics to evaluate the antidepressant activity of green kiwifruit in mice and to identify the fruit bioactive phytochemicals responsible for this effect. Mice treated with green kiwifruit juice showed dose-dependent reductions in depressive behavior. Two kiwifruit-derived metabolites – quinic acid and caffeic acid sulfate (the latter formed in mice via metabolism of kiwifruit caffeic acid glucosides) – were detected in mouse serum and brain tissue, suggesting they may confer the observed effects. When administered as pure compounds, quinic acid closely replicated the antidepressant effect of kiwifruit juice, whereas caffeic acid glucoside had little impact. Other fruit metabolites may act synergistically with quinic acid to increase its bioavailability in serum and its absorption into the brain parenchyma. Our approach thus led to the discovery of quinic acid as the kiwifruit metabolite capable of rapidly reaching the mouse brain and exerting an antidepressant effect in synergy with other fruit metabolites.

## Introduction

The beneficial effects of a diet rich in fruits, vegetables, nuts, and other plant-based ingredients are well known and recognized, especially for the prevention of cardiovascular disease [1,2]. More recently, links have emerged between plant-based diets and mental health. Nutrition and mental health are generally interconnected and reciprocally affected, and this has been observed primarily with depression [3–5]. Healthy nutrition plays a key role in the onset and severity of some mental health disorders, with affected individuals frequently showing nutritional deficiencies [6].

Observational and small interventional studies have investigated the effects of a diet rich in plant-based ingredients or the entire dietetic patterns containing them. One example is the Mediterranean diet, typical in olive-growing regions of the Mediterranean [7,8], which has been associated to cognitive performance benefits [9,10], the prevention of depression [11–14] and general psychological wellbeing [15–20]. Randomized controlled interventional studies [21], as well as in vitro and in vivo tests in animal models and humans, suggest that some fresh and even processed fruits and vegetables have positive effects on the brain, including gold kiwifruit (*Actinidia chinensis*) [22], green kiwifruit (*Actinidia deliciosa*) [23], blackcurrant, blueberry, cherry, cranberry, grape, apple [24], orange juice [25,26], and onion [27]. However, the active compounds and their effectiveness in vivo remain largely unknown.

The active compounds in complex mixtures, such as natural phytocomplexes, can be difficult to find. Traditional bioassay-guided fractionation is a powerful approach, but the fractionation procedures are time-consuming and tend to dilute the active components as well as separating them from potentially synergistic compounds. Such limitations can be addressed by metabolomics and biochemometrics [28,29]. Furthermore, the investigation of compound bioavailability recently led to the identification of active molecules (and their metabolic products in mice) in a brain-bioactive dietary polyphenol preparation composed of different grape ingredients, revealing their molecular targets and mechanisms of action [30].

Here we applied untargeted metabolomics to kiwifruit juice, mouse blood and brain tissue to identify brain-bioactive compounds present in green kiwifruit. One molecule (quinic acid) was responsible for most of the observed anti-depressant effects of the whole juice, but other metabolites may act as co-factors to increase its levels in the serum and brain parenchyma. Our two-step approach, combining untargeted metabolomics with in vivo bioassays, can help uncover bioactive compounds in complex plant mixtures, providing insight into their interactions with each other and with targets in vivo.

## Materials and methods

### Chemicals

Fluoxetine HCl was purchased from Alomone Laboratories (Jerusalem, Israel. Cat. No.: F155), and escitalopram from MedChemExpress (Monmouth Junction, NJ, USA. Cat. No.: HY-14258). d-(−)-quinic acid (QA) and caffeic acid 3β-d-glucoside (CAG) were purchased from Santa Cruz Biotechnology (Heidelberg, Germany). Acetonitrile, methanol and water were purchased from Honeywell (Seezle, Germany), formic acid from Biosolve (Dieuze, France) and leucine-enkephalin solution from Waters (Milan, Italy). All solvents used were LC-MS grade.

### Animal treatment

Naïve male C57Bl/6JOlaHsd mice (Envigo RMS Srl, San Pietro al Natisone, Udine, Italy), 5 weeks of age and weighing 20–25 g, were housed in groups of six in Optimice cages (36.3 × 29.2 × 15.5 cm) with sawdust as a bedding material. The temperature was maintained at 21 ± 1 °C, the relative humidity at 60%, and we imposed a 12-h photoperiod from 7:00–19:00. Food (Mucedola NFM18) and water were provided ad libitum. Animals were allowed to adapt to laboratory conditions for at least 2 weeks before experiments. They were randomly assigned to experimental or control groups (n = 12/16 for behavioral experiments, n = 4 for pharmacokinetics and n = 6 for perfusion). Procedures involving animals were carried out at the University of Verona according to the Italian Governing Law (D.lgs 26/2014; authorization no. 19/2008-A issued 6 March 2008 by the Ministry of Health), the NIH Guide for the Care and Use of Laboratory Animals (2011 edition), and EU Directive 2010/63/EU. All reported animal care and experimental procedures were approved by the ethical committee (OPBA) of the University of Verona and by the Ministry of Health (authorization nos. 775/2016-PR and 68/2020-PR).

### Preparation and administration of kiwifruit juice and vehicle solutions

Kiwifruits (*Actinidia deliciosa* cv. Hayward) were sourced from local producers. The fruits were immediately peeled, sliced and frozen in liquid nitrogen. The frozen material was powdered using an A11 basic analytical mill (IKA-Werke, Staufen, Germany) and the powder was stored at –80 °C. To prepare the kiwifruit juice, 15 g of frozen homogenized powder were thawed and centrifuged at 3,650 × *g* for 15 min at 4 °C. The supernatant was centrifuged again at 21,000 × *g* for 15 min at 4°C and passed through a 0.22-µm Millex PES filter (MilliporeSigma, Milan, Italy). This undiluted juice (Kiwi 1) was further diluted 1:2 (Kiwi 2) and 1:3 (Kiwi 3) with MilliQ water (MilliporeSigma). To exclude the effect of bulk primary metabolites (e.g., sugars, organic acids and ascorbic acid) on the behavioral tests, we created a vehicle solution replicating the concentrations of the major components as previously described [31].

Mice were treated with the three kiwifruit juice preparations or vehicle solution for 10 days using an intragastric (IG) gavage method (as a volume of 10 mL/Kg; [32]) and were tested 90 min after the last dose. In positive controls, fluoxetine or escitalopram were administered intraperitoneally (IP) 30 min before the test at concentrations of 20 and 10 mg/kg, respectively [33,34].

### Behavioral tests

For the forced swim test (FST), mice were placed for 6 min in a transparent poly(methyl methacrylate) cylinder (46 cm height × 20 cm diameter) filled to 30 cm with water at 25 ± 1 °C. Tank dimensions prevented mice from touching the bottom with their paws or tails during the test. Only the last 4 min of the test were analyzed because most mice tend to be more active at the beginning of the FST [35]. The immobility time was manually scored by a trained observer.

For the tail suspension test (TST), mice were suspended by the tail using a 17-cm tape attached to a fixed grid 60 cm above the bench [36,37]. To prevent tail-climbing behavior commonly observed in C57Bl/6J strain mice [38], which can confound the assessment of immobility times, a climb-stopper (4 cm length, 1.6 cm outer diameter, 1.3 cm inner diameter, 1.5 g) was used. Immobility time was manually scored by a trained observer.

In both tests, one rectangular wood divider was used between tanks to prevent mice from seeing each other and potentially altering their behavior. A white background enhanced contrast between the mice and wall in the recorded videos. Data represent the combined results of two independent experiments performed at different times using separate groups of animals to ensure reproducibility.

For the open field test (OFT), the locomotor activity of the mice was assessed for 5 min in a 40 × 40 cm open square arena with low light (~40 lux). Each mouse was taken from its cage and allowed to acclimatize for at least 1 h before the test. Total distance traveled and mean velocity of each mouse were recorded and analyzed using MATLAB Toolbox [39].

### Preparation of mice serum and brain samples

After the behavioral test, mice were euthanized for serum and brain collection. Under deep isoflurane anesthesia, blood was drawn from the retro-orbital sinus using a non-heparinized glass capillary, left to clot at room temperature for 20 min, then centrifuged at 6,800 × *g* for 5 min at 4 °C. Supernatants were frozen in liquid nitrogen and stored at –80° C. Brains were dissected, washed in 0.9% saline, frozen in liquid nitrogen, and stored at –80°C. Perfusion was carried out by washing the entire blood volume of anesthetized mice with cold 0.9% saline for 5 min [40], then dissecting and freezing the brains as described above.

### Metabolite extraction for targeted and untargeted UPLC-qTOF-MS analysis

Serum samples were thawed at room temperature and 1-mL aliquots were diluted with 10 volumes of cold LC-MS-grade methanol. After mixing for 30 s and centrifuging at 3,650 × *g* for 15 min at 4 °C, the supernatant was transferred to a fresh tube and centrifuged at 21,000 × *g* for 20 min at 4 °C. The supernatant was passed through an Oasis PRiME HLB 1 cc Vac cartridge containing 30 mg of sorbent (Waters) attached to a Waters 20-position extraction manifold to remove proteins and phospholipids, according to the manufacturer’s instructions.

For metabolomics analysis, the methanol extracts were diluted 1:2 with LC-MS-grade water for C18 analysis and 1:2 with LC-MS grade methanol for HILIC analysis. Extracts were then passed through 0.2-μm Minisart RC4 filters (Sartorius, Göttingen, Germany) and 3 μL of each extract was injected into the UPLC-qTOF-MS system.

Brain samples (about 180–220 mg) were homogenized in 10 volumes (w/v) of LC-MS-grade methanol at 4 °C using a Precellys cryolys evolution (Bertin, Montigny-le-Bretonneux, France), then sonicated at 40 kHz in a Sonica Ultrasonic Cleaner ultrasonic bath (SOLTEC, Milano, Italy) for 20 min. This was followed by two rounds of centrifugation, first at 3,650 × *g* for 15 min and then at 21,000 × *g* for 15 min (both at 4° C). The supernatant was passed through an Oasis PRiME HLB 1 cc Vac cartridge for UPLC-MS as described above.

For the analysis of kiwifruit juice, 100 µL of Kiwi 1 was mixed with 900 µL of LC-MS-grade methanol at –20 °C and centrifuged at 21,000 × *g* for 10 min at 4 °C. The supernatant was diluted and filtered as above, and 1 μL was injected in the UPLC-qTOF-MS system.

### UPLC-qTOF-MS untargeted metabolomics

A Waters ACQUITY I CLASS UPLC system equipped with a refrigerated autosampler was connected to a Waters eLambda 800 nm PDA detector and a Waters Xevo G2-XS qTOF mass spectrometer featuring an electrospray ionization (ESI) source operating in either positive or negative ionization mode. The system was controlled by MassLynx v4.1. All extracts were injected into Waters ACQUITY UPLC BEH C18 and HILIC columns (2.1 mm × 100 mm, 1.7 μm) kept at 30 °C. The C18 mobile phases consisted of 0.1% formic acid in water (A) and acetonitrile (B). The initial conditions were 99% A and 1% B and the following elution gradient was applied: 0–1 min, 1% B; 1–10 min, 1–40% B; 10–13.50 min, 40–70% B; 13.50–15.00 min, 70–90% B; 15.00–16.50 min, 90–100% B, 16.50–20 min, 100% B, 20–20.1 min, 100–1% B (initial conditions). The system was then equilibrated in 99% A up to 25 min. The HILIC mobile phases consisted of 20 mM ammonium formate in water (A) and 5% 10 mM of ammonium formate in water plus 95% acetonitrile (B). The initial conditions were 0% A and 100% B, and the following elution profile was applied: 0–3 min, 100% B; 3–7 min, 100–85% B; 7–10 min, 85% B; 10–15 min, 85–50% B; 15–20 min, 50% B; 20–20.10 min, 50–100% B (initial conditions). The system was then equilibrated in 100% B up to 30 min. The flow rate was set to 0.350 mL/min for both columns. Samples were kept at 8 °C and analysis was randomized. A quality control (QC) sample was prepared by mixing equal parts of all samples in order to check UPLC-qTOF-MS performance. QC was injected after nine samples had been analyzed. Ion source parameters: capillary voltage 0.8 kV, sampling cone voltage 40 V, source offset voltage 80 V, source temperature 120 °C, desolvation temperature 500 °C, cone gas flow rate 50 L/h and desolvation gas flow rate 1000 L/h. Nitrogen gas was used for the nebulizer and desolvation, whereas argon was used to induce collision-induced dissociation. An MS method was created to acquire data in continuum mode using a fixed collision energy in two scan functions. In function 1 the low energy was disabled, whereas in function 2 the high energy was set to 35 V. For some samples, the high energy was increased to 45 V to achieve the better fragmentation of certain metabolites. In both functions, the Xevo G2-XS was set to perform the analysis in sensitivity mode, within the range 50–2000 *m*/*z* and with a scan time of 0.3 s. The lock mass solution used as “calibrator” to verify the accuracy of the mass spectrometer consisted of a 100 pg/μL leucine-enkephalin solution injected at a flow rate of 10 μL/min, generating a signal of 556.2771 *m/z* in positive mode and 554.2615 *m/z* in negative mode.

### Quinic acid and caffeic acid glucosides quantification in fresh kiwifruit juice

The absolute quantification of metabolites by LC-MS requires a careful evaluation of potential matrix effects that cause ion suppression or enhancement. Kiwi 1 was sequentially diluted, and the matrix effect disappeared at a dilution of 1:1600 for QA and 1:100 for CAG, but both metabolites were still well detectable. We also confirmed the absence of matrix effects by spiking and comparing the peak areas of three groups of samples: (1) diluted kiwifruit juice alone, (2) the same kiwifruit juice spiked with 1.5 ng/µL QA and 125 pg/µL CAG, and (3) a solution of pure standard compounds at the same concentrations. We analyzed 1 µL of each diluted solution three times by UPLC-qTOF-MS and no matrix effect was found for either of the metabolites. The peak area of the metabolites of interest was normalized for the dilution factor and compared with a calibration curve obtained using QA and CAG authentic standards.

### Data processing and metabolite identification

The statistical significance of behavioral data was analyzed using Prism v9.0 (GraphPad Software, San Diego, CA, USA). Significant differences between samples were determined by one-way analysis of variance (ANOVA) followed by Dunnett’s post hoc test. The raw untargeted metabolomics data were processed using Progenesis QI (Waters). Absolute ion intensity for peak picking was set at 300, with a minimum chromatographic peak width of 0.03 min. An in-house library of authentic reference standards was used for metabolite identification, by comparing retention time, *m*/*z* ratios, isotope similarities, fragmentation patterns (MS/MS) and UV-Vis absorbance spectra. Further tentative identifications were achieved using Metlin (https://metlin.scripps.edu) with a tolerance of 0.003 Da and an automatic online search of public databases (MassBank, PlantCyc, Plant Metabolic Network and Human Metabolome Database). Finally, literature data were used to support the putative annotations. QA and CAG identities were confirmed with authentic standards; the *m/z*, fragmentation pattern (MS/MS) and the UV-Vis absorbance spectrum of CAG were used also to identify its structural isomers. Because internal standards were not used, relative quantitation (i.e., comparison between samples) was based on the area of each of the signals extracted from the chromatograms and expressed in arbitrary intensity units. Orthogonal two‐block partial least squares-discriminant analysis (O2PLS-DA) was applied to the metabolomics feature quantification matrices using SIMCA 13.0 (Umetrics) after Pareto scaling and centering. A permutation test (200 permutations) was used for each OPLS-DA model to avoid overfitting.

## Results

### Kiwifruit juice shows dose-dependent antidepressant activity in mice

**Fig 1:**
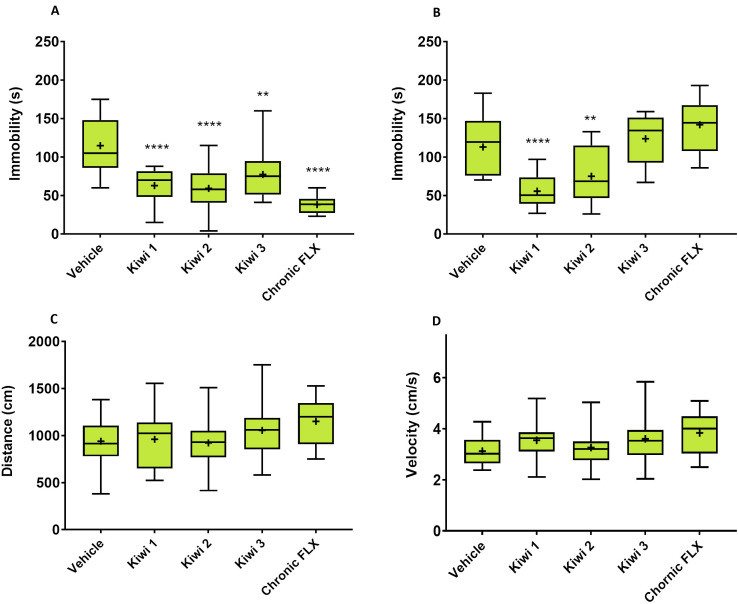
Dose-dependent antidepressant activity of fresh kiwifruit juice preparations in mice. The effect of undiluted (Kiwi 1) and diluted (Kiwi 2 and Kiwi 3) kiwifruit juice preparations was tested by measuring the immobility time in the TST (A) and FST **(B)**, and the distance traveled (C) and mean velocity (D) of mice in the OFT. Statistical significance was determined by one-way ANOVA followed by Dunnett’s post hoc test. Data are means ± SD (n = 12/16 per group; **p ≤ 0.01, ****p ≤ 0.0001 vs. vehicle-treated group). FLX = fluoxetine.

The antidepressant activity of fresh kiwifruit juice preparations (undiluted Kiwi 1 and its dilutions Kiwi 2 and Kiwi 3) was investigated in mice using the tail suspension test (TST) and forced swim test (FST) 90 min after the final intragastric (IG) administration in a 10-day trial. The highest dose (10 mL/kg Kiwi 1) was equivalent to the consumption of ~7 fruits by a 70-kg human male. Intraperitoneal (IP) fluoxetine (Prozac) at 20 mg/kg was used as a positive control. A vehicle solution containing all the bulk primary metabolites of kiwifruit juice in the same doses found in the fruit was used as a negative control, to which all treatment groups were compared. Kiwi 1 strongly reduced the duration of immobility in both tests compared to the vehicle-treated group (F(6,88)_TST_ = 15.39, p ≤ 0.0001; F(6,88)_FST_ = 11.79, p ≤ 0.0001), whereas Kiwi 2 and 3 were less active, with Kiwi 3 showing minimal effect in the FST (Fig 1A, 1B). Fluoxetine also had no effect on the FST as reported in the literature with this strain of mice [33,41]. Locomotor activity was evaluated in the open field test (OFT) on day 8 to assess locomotor impairment, but there was no significant difference between any of the groups (Fig 1C, 1D).

### Few specific kiwifruit-derived metabolites are present in post-treatment mouse serum and brain tissue

**Fig 2:**
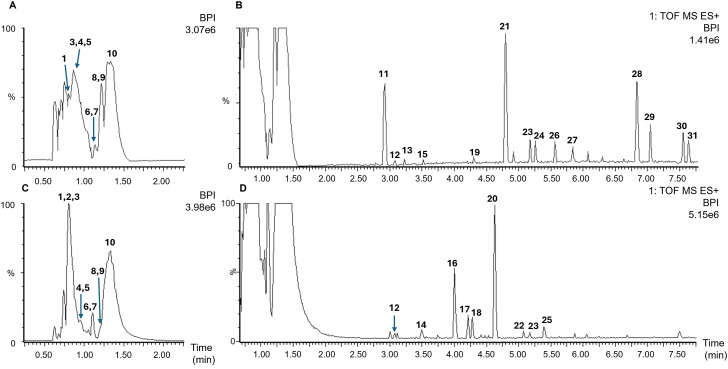
The main metabolites present in fresh kiwifruit juice (Kiwi 1). UPLC-qTOF-MS chromatograms in positive ionization (A,B) and negative ionization (C,D) mode reveal metabolites eluting in the first 2 min (A,C) and other metabolites **(B,D)**, shown on a different scale. The following metabolites are enumerated: 1, quinic acid; 2, dihexose-deoxyhexose; 3, sucrose; 4, malic acid; 5, ascorbic acid; 7 and 9, quinic acid derivatives; 8, glutathione; 10, citric acid; 11, serotonin; 13, phenylalanine; 14, caffeoyl diglucoside; 16, caffeic acid glucoside; 18, esculetin-6-D-glucoside; 20, caffeic acid-3-β-D-glucoside; 21, tryptamine; 22, fraxin; 25, epicatechin. Other peak numbers were unidentified. For all UPLC-qTOF-MS features, see S1 Table.

The fresh kiwifruit juice (Kiwi 1) was analyzed by UPLC-qTOF-MS with an untargeted metabolomics approach. The complex mixture was predominantly composed of primary metabolites such as organic acids and sugars, with lower levels of secondary metabolites such as caffeic acid derivatives, flavonoids and indoleamines (Fig 2; S1 Table). Many other low-abundance metabolites were also detected, some of which could not be identified (S1 File).

**Fig 3:**
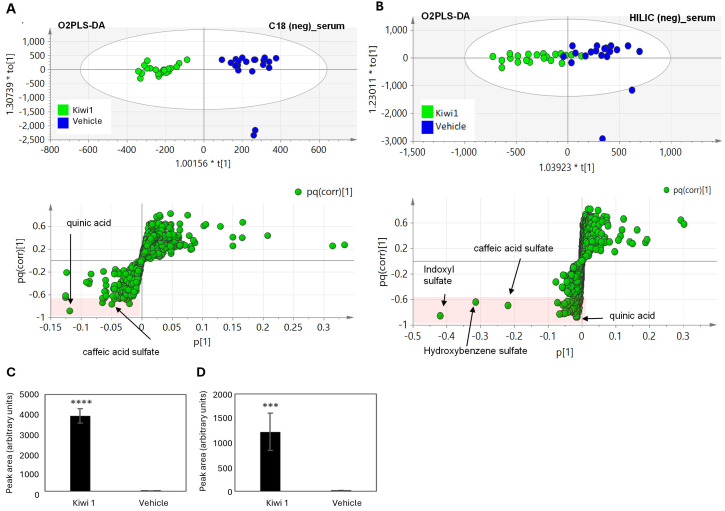
Untargeted metabolomic analysis of mouse serum and brain tissue following the IG administration of kiwifruit juice (Kiwi 1). A,B) O2PLS-DA overview score plot and S-loading plot of serum samples analyzed in negative ionization mode following separation on C18 and HILIC columns (each dot represents one sample in the score plots or one metabolite in the S-loading plots). C,D) Relative amounts of QA and CAS in the brain 30 min after the final administration of Kiwi 1. Data are means ± SD (n = 12/16 per group). Statistical significance was evaluated using Student’s t-test (*p ≤ 0.05, **p ≤ 0.01, ***p ≤ 0.001 ****p ≤ 0.0001).

To identify candidate molecules responsible for the observed antidepressant effect, we investigated the bioavailability of kiwifruit metabolites in mouse serum and brain tissue at different time points after administration. The Kiwi 1 treatment group was compared with vehicle-treated control, and multivariate analysis of the metabolic feature quantification matrix was used to find differences between the groups. We applied two forms of chromatography (HILIC and C18 column chemistry; S2 File) to include as many metabolites as possible ranging from high to low polarity. Only two kiwifruit metabolites were identified in the serum and brain tissues: quinic acid (QA), which is abundant in kiwifruits, and caffeic acid sulfate (CAS), probably generated when caffeic acid-based molecules in kiwifruit (caffeic acid glucosides and esters) are metabolized in mice (Fig 3A, 3B). Other metabolites in the serum of kiwifruit-treated mice were not present in the kiwifruit juice itself (e.g., indoxyl and hydroxybenzene sulfates). These are likely to be products formed when kiwifruit compounds are metabolized in mice, or endogenous mouse metabolites that accumulate in response to kiwifruit juice consumption (S2 Table). In brain samples, the same analysis showed limited statistical significance, probably due to the very low level of kiwifruit metabolites found in brain tissues. However, both QA and CAS could be manually extracted from the raw chromatograms (Fig 3C, 3D).

Kiwifruit contains relevant amounts of tryptamine (a serotonin agonist in humans) and ~0.5 mg per 100 g fresh weight of serotonin [42]. We therefore tested the bioavailability of these indolamines following the oral administration of tryptamine and deuterated serotonin (to distinguish it from the endogenous molecule). Neither of these compounds was detected in the serum or brain of treated mice.

### Quinic acid mimics the effects of kiwifruit juice

**Fig 4:**
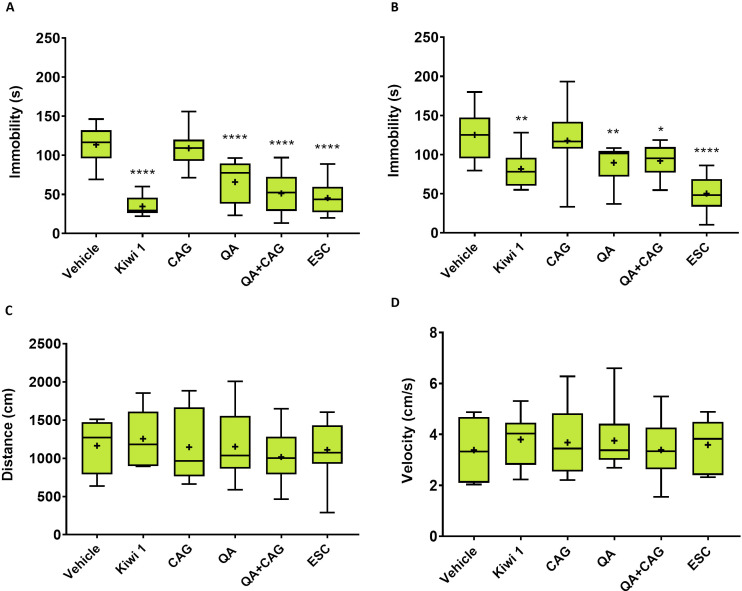
Antidepressant effects of the single and combined administration of QA and CAG in relation to kiwifruit juice. The effect of kiwifruit juice (Kiwi 1), vehicle, QA, CAG and their combination (QA + CAG) was assessed by measuring the immobility time in the TST (A) and FST **(B)**, and the distance travelled (C) and mean velocity (D) in the OFT. Data are means ± SD (n = 12 per group). Statistical significance was evaluated by one-way ANOVA followed by Dunnett’s post hoc test (* p ≤ 0.05, **p ≤ 0.01, ****p ≤ 0.0001 vs. vehicle-treated group). ESC = escitalopram.

Given that only two compounds (QA and CAS) found in mouse brain tissue were traceable to specific kiwifruit molecules, we decided to test QA and one caffeic acid glucoside isomer (caffeic acid 3β-D-glucoside, CAG; i.e., the main form of caffeic acid in kiwifruit juice that is metabolized to CAS in mice), individually and in combination, at the same concentrations found in Kiwi 1. We therefore determined the precise quantity of both molecules in kiwifruit juice using a dilution method. We then repeated the 10-day IG trials in mice using these purified components (as well as Kiwi 1, for comparison), followed by TST and FST 90 min after the last treatment on day 10. Given that 20 mg/kg fluoxetine was unsuitable as a positive control in the TST (Fig 1), we replaced it with escitalopram (Cipralex) administered intraperitoneally (IP) 30 min before the test at a concentration of 10 mg/kg [43,44]. As in the previous experiment, Kiwi 1 significantly reduced the duration of immobility in both tests compared to the vehicle-treated group. QA given alone or in combination with CAG, at the same concentrations found in Kiwi 1, also significantly reduced the duration of immobility, albeit not to the same extent as the whole juice. CAG alone had no effect, even taking into account the high inter-subject variability within this group (F(5,66)_TST_ = 26.33, p ≤ 0.0001; F(5,66)_FST_ = 11.54, p ≤ 0.0001) (Fig 4). Escitalopram was a good positive control for both tests. OFT on day 8 again excluded locomotor impairment as an explanation for the results. Accordingly, we concluded that purified QA, administered at the same concentration found in kiwifruit juice, can partially mimic the antidepressant activity of the juice in mice, indicating that QA is one of the bioactive antidepressant components.

**Fig 5:**
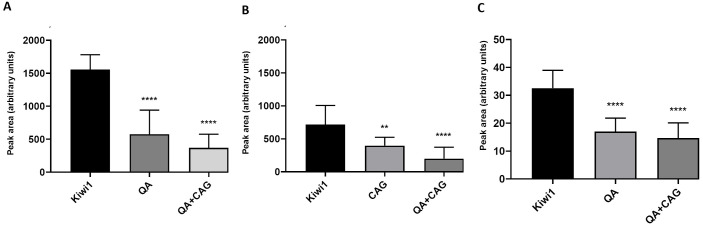
Bioavailability of QA and CAG administered as pure compounds or as components of kiwifruit juice (Kiwi-1). **A)** The relative quantity of QA in serum following separation by C18 chromatography. **B)** The relative quantity of CAG in serum following separation by HILIC. **C)** The relative quantity of QA in brain tissue following separation by C18 chromatography. Data are means ± SD (n = 12 per group). Statistical significance was determined by one-way ANOVA followed by Dunnett’s post hoc test (**p ≤ 0.01, ****p ≤ 0.0001 vs. Kiwi 1-treated group).

Following the behavioral experiments, serum and brain tissue samples were collected and analyzed by UPLC-qTOF-MS. Interestingly, the levels of QA and CAS in serum, and the level of QA in the brain, were lower in all treatments with purified metabolites compared to the levels of the same molecules following treatment with Kiwi 1 (Fig 5). CAS was detected in the brain but was not evaluated because the amount was below the lower limit of quantification. This suggests kiwifruit contains other components that promote the adsorption and/or stability of these molecules in vivo. The lower activity of pure QA compared with Kiwi 1 may thus reflect the activity of other low-level, or even undetectable, kiwifruit molecules or, as suggested by the lower level of QA in QA-treated mice, the limited absorption and/or stability of QA when ingested in its pure form.

**Fig 6:**
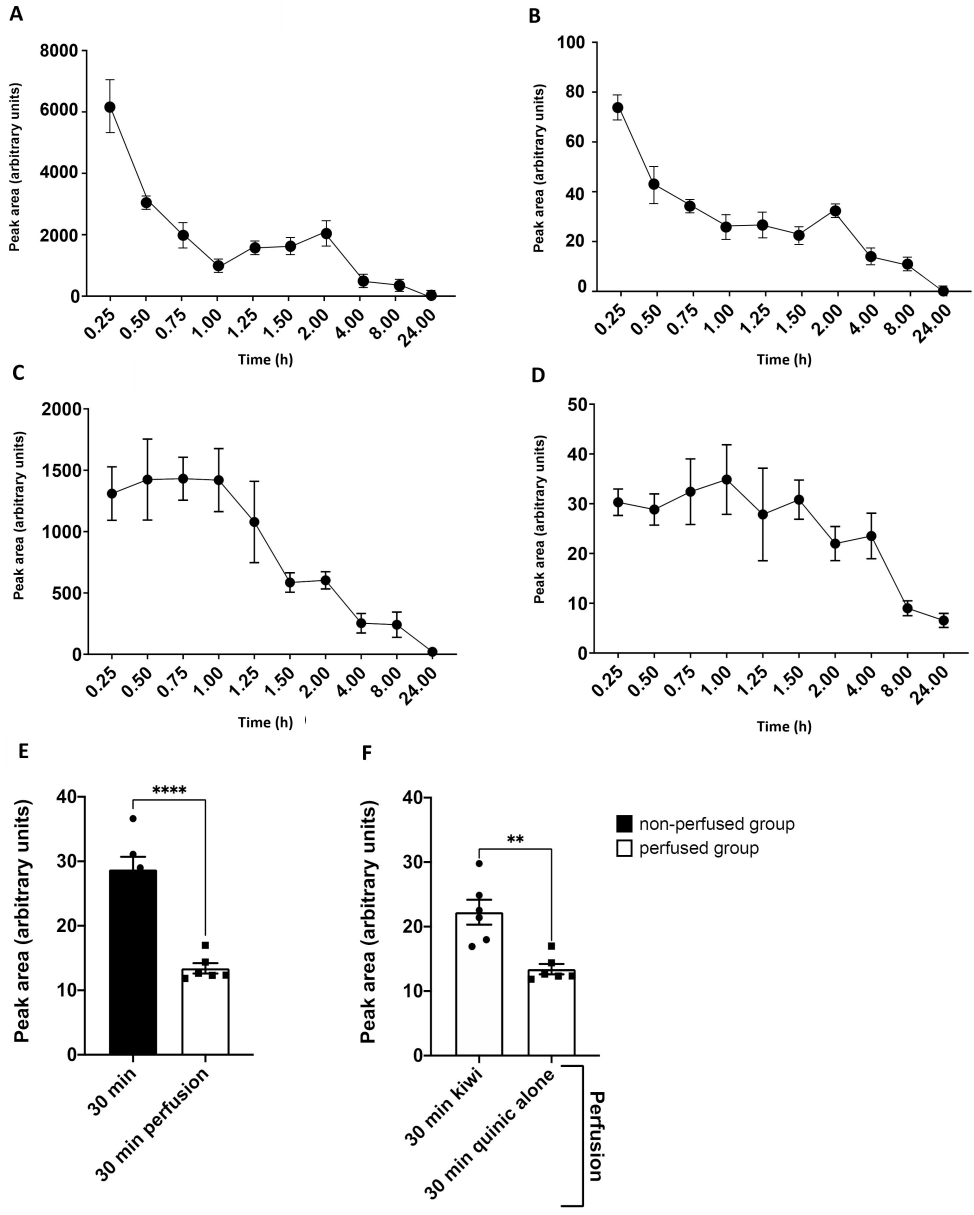
Pharmacokinetics of QA in the serum and brain tissues of mice after kiwifruit (Kiwi-1) or pure QA administration. Relative amount of QA in the serum (A,C) and brain tissue (B,D) of mice following the administration of kiwifruit juice (A,B) or pure compound **(C,D)**. Each time point represents the mean ± SD of n = 4 readings. Relative amount of QA in the brain with and without perfusion (E) and a comparison of perfused animals treated with kiwifruit or with QA 30 min before they were euthanized **(F)**. Data are means ± SD (n = 6 per group). Statistical significance was evaluated using an unpaired t-test (**p ≤ 0.01, ****p ≤ 0.0001).

The pharmacokinetics of QA were determined in mice that were treated with Kiwi 1 (Fig 6A, 6B) or the pure molecule (Fig 6C, 6D) and then euthanized at different time points after administration. We also evaluated the real-time tissue penetration profile of QA in the brain parenchyma by perfusing the brains with cold 0.9% saline for 5 min (Fig 6E, 6F).

As anticipated, QA declined in the brain after perfusion regardless of the treatment group, but was nevertheless detected in both types of sample.

## Discussion

The antidepressant activity of green kiwifruit juice was assessed in mice by applying the TST and FST, two behavioral paradigms widely used to assess depressive-like behavior [45–47] and to evaluate the potency of antidepressant drugs or the antidepressant effects of natural extracts [48]. Both models have long been used in rodents due to their high face, construct and predictive validity [49]. We chose IG administration to precisely control dosage and to mimic the real absorption of phytocomplexes. Kiwifruit juice significantly reduced immobility time in both tests (even compared to standard antidepressants) without locomotor impairment, as confirmed by OFT. We initially used 20 mg/kg fluoxetine as a positive control, a dosage consistent with plasma levels observed in human patients undergoing acute or chronic regimes [41]. However, the drug did not affect FST outcomes, as previously reported for this strain of mice [33]. We therefore switched to escitalopram as a positive control in subsequent experiments.

Previous studies have linked green and gold kiwifruit with antidepressant effects in mice and humans, respectively, although the active compounds have never been identified [22,23]. A recent double-blind registered and approved trial to assess the impact of vitamin C and vitamin C-rich SunGold kiwifruit on mood enhancement revealed that the consumption of two fruits per day improved the mood more effectively than either a placebo or pure vitamin C. This effect was apparent even though the two treatments showed equivalent vitamin C bioavailability, suggesting that additional kiwifruit components contributed to mental health improvements [50].

Other investigations have shown that green kiwifruit improves sleep quality in young adults with sleep disorders [51,52]. Both green and gold kiwifruit contains a relevant amount of serotonin [53] and its plant precursor tryptamine [42]. In principle, serotonin and its derivative melatonin could be involved in sleep improvement [54], even though we did not detect melatonin in our experiments. Tryptamine could also act in the same pathway, as it functions as a serotonin agonist [55,56]. Dietary serotonin and tryptamine are accumulated in many fruits, as plantains, bananas, pineapples, plumes, tomatoes, walnut and peppers [57–59]. The two indolamines are generally not bioavailable because they are broken down by the monoamine oxidases MAO-A and MAO-B in the gut, giving rise to 5-hydroxyindoleacetic acid [53]; however, they may survive in the form of a phytocomplex derived from fruits or vegetables, allowing synergic activity. As an example, the shamanic beer ayahuasca retains the hallucinogenic properties of dimethyltryptamine contained in the herb *Psychotria viridis* thanks to the ability of β-carbolines (harmine, harmaline and tetrahydroharmine) in *Banisteriopsis caapi* to inhibit MAO [60,61]. Similar inhibitory components in kiwifruits could change the fate of fruit-derived serotonin and tryptamine by making them more bioavailable.

Despite the presence of MAO inhibitors in kiwifruit [31], fruit-derived serotonin and tryptamine were not present in the blood or brain of mice fed on kiwifruit juice. However, quinic acid (QA) was present in the blood and (at lower levels) in the brain, confirming its ability to cross the blood–brain barrier. TST and FST results confirmed the antidepressant effects of this compound. Caffeic acid sulfate (a metabolic breakdown product of the caffeic acid glucosides found in kiwifruit) was also present, but did not significantly influence behavior in the TST and FST. Not all the activity of fruit juice could be explained by QA because the administration of the pure molecule at the same concentration found in kiwifruit juice was less effective than whole fruit juice. This suggests there is a synergistic effect involving QA and other metabolites or juice components that increase its bioavailability and/or activity. As recently revised [62], various plant components influence the bioavailability of secondary metabolites. For instance, proteins, dietary fiber, and minerals negatively affect the bioavailability of flavonoids, while lipids, carbohydrates, vitamins, alkaloids, carotenoids and other flavonoids enhance it. The juice used for our experiments likely contained numerous components beyond the metabolites detected and described in this work, including proteins, soluble carbohydrates, minerals, and vitamins, while insoluble carbohydrates were probably eliminated by centrifugation. Any of these remaining components could potentially influence the bioavailability of QA. Subsequent experiments showed that the accumulation of QA, administered as a component of kiwifruit juice or a pure molecule, was very rapid, that QA was able to cross the blood–brain barrier and penetrate the brain parenchyma, and that the amount of QA in the serum and brain was lower when administered as pure molecule or in combination with caffeic acid 3β-D-glucoside (CAG). Thus, the lower activity of pure QA compared to fruit juice in the TST and FST can largely be attributed to its reduced ability to reach the brain parenchyma.

The effects of individual metabolites in fresh fruits and vegetables are difficult to unravel, not only due to the large number of different molecules but also because their activity could depend on additive, synergic and antagonist effects involving other compounds [63,64]. Many factors could also influence the bioavailability of metabolites, as is the case of dimethyltryptamine in ayahuasca beer [60,61]. Synergy and antagonism are difficult to study rigorously, so the development of drugs from natural products tends to focus on reducing complex mixtures to single bioactive components [65]. However, synergies among combinations of phytochemicals can promote the solubility, safety, absorption, stability and/or bioavailability of bioactive compounds [64]. Antagonism, which has been investigated in less detail, causes the effects of active constituents to be masked by other compounds in a complex mixture. Such synergistic and antagonistic relationships may explain why no single compound can replace the combination of natural phytochemicals in fruits and vegetables to achieve the same health benefits [63]. We found that QA administered alone, at the same concentration of the kiwifruit juice, had less activity than undiluted juice, correlating with the lower levels detected in serum and brain samples. This suggests that the whole phytocomplex could be important for stability and/or adsorption but not necessarily for activity.

QA was discovered in the medicinal plant *Cinchona officinalis* [66] and is synthesized from the shikimic acid precursor 3-dehydroquinate. Free QA is uncommon in plants, but accumulates mainly in fruits, whereas most tissues contain QA esterified with other secondary metabolites, especially hydroxycinnamic acids such as caffeic acid [67]. Accordingly, the bioactive properties of free QA are rarely discussed [68] whereas the effects of quinic acid esters are well characterized [67,69], including activities that influence neuroprotection, cognition and mood in animal models [70–72]. In humans, caffeic acid esters of QA are absorbed partially as intact molecules and partially after hydrolysis in the stomach and small intestine [67,69]. The metabolites released by hydrolysis are metabolized further by the gut microbiome as well as mammalian phase II metabolism [73]. The hydrolysis and microbial metabolism of caffeoylquinic acid has also been described in rats [74]. In our experimental mice, we found that caffeic acid derived from CAG was sulfated and inactive, whereas free QA – detected in the brain parenchyma – retained the activity. Its release from more abundant dietary esters [69] suggests that QA could be, at least partially, responsible for the many bioactivities typically attributed to these esters, including the ubiquitous chlorogenic acids.

## Supporting information

S1 Table Main metabolites of kiwifruit fresh juice putatively identified or unidentified. The IDs are as shown in Fig 2. RT = retention time; UI = unidentified; *identification confirmed using authentic standards.
(DOCX)

S2 Table Metabolites found in the serum of kiwifruit-treated mice. RT = retention time; *identification confirmed using authentic standards.
(DOCX)

S1 File Other metabolites in fresh kiwifruit juice. These lists integrate the metabolites shown in Fig 2 and S1 Table. Putatively identified (a) and unidentified (b) metabolites detected in negative ionization mode. RT = retention time, *identification confirmed using authentic standards.
(XLSX)

S2 File Minimal datasets of the experiments. Data collected from the untargeted metabolomics analysis and the in vivo experiments.
(XLSX)
