# Supplementary material for: Untargeted metabolomics reveals quinic acid as the kiwifruit component that affects brain activity in mice
Source: PLoS One. 2025 Aug 18;20(8):e0326134. doi: 10.1371/journal.pone.0326134 (PMC12360534; doi:10.1371/journal.pone.0326134)
Supplement: S1 Table — The IDs are as shown in Fig 2. RT = retention time; UI = unidentified; *identification confirmed using authentic standards. (DOCX) [file pone.0326134.s001.docx]

**Untargeted metabolomics reveals quinic acid as the kiwifruit component that affects brain activity in mice – Supplementary Tables**

Claudio Marcelo Marzo, Martino Bianconi, Mauro Commisso, Sofia Gambini, Cristiano Chiamulera, Linda Avesani, Stefano Negri and Flavia Guzzo

Corresponding authors: Flavia Guzzo and Stefano Negri (Department of Biotechnology, University of Verona, Strada Le Grazie 15, 37134, Verona, Italy). E-mails: [flavia.guzzo@univr.it](mailto:flavia.guzzo@univr.it); [stefano.negri@univr.it](mailto:stefano.negri@univr.it)

**Supplementary Table 1: Main metabolites of kiwifruit fresh juice putatively identified or unidentified (UI). The IDs are as shown in Figure 2. RT = retention time; *identification confirmed using authentic standards.**

| **ID** | **RT (min)** | **Elemental formula** | **Putative identification** | **(-) Ion** | ***m/z* (-) detected** | ***m/z* (-) expected** | **Mass error (ppm)** | **Main fragments (-)** | **(+) Ion** | ***m/z* (+) detected** | ***m/z* (+) expected** | **Mass error (ppm)** | **Main fragments (+)** |
| --- | --- | --- | --- | --- | --- | --- | --- | --- | --- | --- | --- | --- | --- |
| **1** | 0.803 | C_7_H_12_O_6_ | quinic acid* | [M-H^+^]^-^ | 191.056 | 191.056 | -0.661 | 108.02; 111.043; 127.038; 173.044 | [M+Na^+^]^+^ | 215.052 | 215.053 | 4.650 |  |
| **2** | 0.824 | C_18_H_32_O_15_ | dihexose-desoxyhexose |  | 533.173 | 533.171 | -3.035 | 179.055; 341.109 |  | - |  |  |  |
| **3** | 0.845 | C_12_H_22_O_11_ | sucrose | [M+HCOOH-H^+^]^-^ | 387.115 | 387.113 | -3.913 | 179.055; 341.109 | [M+Na^+^]^+^ | 365.105 | 365.105 | 0.000 |  |
| **4** | 0.948 | C_4_H_6_O_5_ | malic acid | [M-H^+^]^-^ | 133.014 | 133.013 | -4.316 | 115.002 | [M+Na^+^]^+^ | 157.012 | 157.011 | -6.369 |  |
| **5** | 0.979 | C_6_H_8_O_6_ | ascorbic acid | [M-H^+^]^-^ | 175.024 | 175.024 | -0.004 | 115.001 | [M+H^+^]^+^ | 177.040 | 177.039 | -5.648 |  |
| **6** | 1.084 |  | UI |  | 605.194 |  |  | 151.060; 173.044; |  | 629.190 |  |  |  |
| **7** | 1.105 |  | quinic acid derivative |  | 391.066 |  |  | 191.053; 217.050; |  | 393.086 |  |  |  |
| **8** | 1.189 | C_10_H_17_N_3_O_6_S | glutathione | [M-H^+^]^-^ | 306.076 | 306.076 | -0.805 | 143.0430 | [M-NH_3_+H^+^]^+^ | 308.092 | 308.091 | -3.246 | 84.044; 116.017; 130.051; 162.021 |
| **9** | 1.199 |  | quinic acid derivative |  | 391.070 |  |  | 191.053; 217.050; |  | 393.086 |  |  |  |
| **10** | 1.333 | C_6_H_8_O_7_ | citric acid | [M-H^+^]^-^ | 191.019 | 191.019 | -0.056 | 111.007; | [M+Na^+^]^+^ | 215.019 | 215.017 | -10.371 |  |
| **11** | 2.810 | C_10_H_12_N_2_O | serotonin* |  | - |  |  |  | [M-NH_3_+H^+^]^+^ | 160.076 | 160.076 | 1.499 | 115.053; 117.058; 132.079; 142.063; 177.098 |
| **12** | 3.095 |  | UI |  | 351.128 |  |  |  |  | 353.147 |  |  | 119.048; 136.074 |
| **13** | 3.238 | C_9_H_11_NO_2_ | phenylalanine* |  | - |  |  |  | [M+H^+^]^+^ | 166.087 | 166.087 | -1.204 | 120.079 |
| **14** | 3.512 | C_21_H_28_O_14_ | caffeoyl diglucoside |  | 549.145 |  |  | 135.045; 179.034; 341.087 |  | - |  |  |  |
| **15** | 3.523 |  | UI |  | 316.150 |  |  |  |  | 318.165 |  |  | 163.038 |
| **16** | 4.023 | C_15_H_18_O_9_ | caffeic acid glucoside | [M-H^+^]^-^ | 341.087 | 341.087 | 1.255 | 135.043; 179.032 |  | - |  |  |  |
| **17** | 4.231 |  | UI |  | 323.133 |  |  |  |  | - |  |  | - |
| **18** | 4.304 | C_15_H_16_O_9_ | esculetin 6-D-glucoside (aesculin)* | [M-H^+^]^-^ | 339.070 | 339.072 | 6.131 | 105.036; 133.031; 177.019 | [M+H^+^]^+^ | 341.087 | 341.087 | 0.733 |  |
| **19** | 4.300 |  | UI |  | 373.208 |  |  |  |  | 375.223 |  |  | - |
| **20** | 4.647 | C_15_H_18_O_9_ | caffeic acid 3-β-D-glucoside* | [M-H^+^]^-^ | 341.087 | 341.087 | 1.539 | 135.043; 179.034 | [M+Na^+^]^+^ | 365.085 | 365.085 | -0.411 |  |
| **21** | 4.769 | C_10_H_12_N_2_ | tryptamine* |  | - |  |  |  | [M-NH_3_+H^+^]^+^ | 144.082 |  |  | 115.054; 117.057; 127.052 |
| **22** | 5.096 | C_16_H_18_O_10_ | fraxin* | [M-H^+^]^-^ | 369.081 | 369.082 | 3.932 | 163.004; 190.997; 192.005; 207.029 | [M+Na^+^]^+^ | 393.080 | 393.080 | -0.611 |  |
| **23** | 5.159 |  | UI |  | 449.237 |  |  |  |  | 451.256 |  |  | 143.119 |
| **24** | 5.264 |  | UI |  | 391.193 |  |  |  |  | 393.210 |  |  | - |
| **25** | 5.410 | C_15_H_14_O_6_ | epicatechin* | [M-H^+^]^-^ | 289.070 | 289.071 | 5.516 | 109.030; 123.045; 137.024; 245.078; 289.071 | [M+H^+^]^+^ | 291.086 | 291.087 | 2.954 |  |
| **26** | 5.566 |  | UI |  | - |  |  |  |  | 437.238 |  |  | - |
| **27** | 5.838 |  | UI |  | - |  |  |  |  | 481.261 |  |  | - |
| **28** | 6.830 |  | UI |  | - |  |  |  |  | 260.175 |  |  | - |
| **29** | 7.047 |  | UI |  | - |  |  |  |  | 420.241 |  |  | 107.05 |
| **30** | 7.556 |  | UI |  | - |  |  |  |  | 420.241 |  |  | 107.05 |
| **31** | 7.640 |  | UI |  | - |  |  |  |  | 420.241 |  |  | 107.05 |
