# Supplementary material for: Untargeted metabolomics reveals quinic acid as the kiwifruit component that affects brain activity in mice
Source: PLoS One. 2025 Aug 18;20(8):e0326134. doi: 10.1371/journal.pone.0326134 (PMC12360534; doi:10.1371/journal.pone.0326134)
Supplement: S2 Table — RT = retention time; *identification confirmed using authentic standards. (DOCX) [file pone.0326134.s002.docx]

**Untargeted metabolomics reveals quinic acid as the kiwifruit component that affects brain activity in mice – Supplementary Tables**

Claudio Marcelo Marzo, Martino Bianconi, Mauro Commisso, Sofia Gambini, Cristiano Chiamulera, Linda Avesani, Stefano Negri and Flavia Guzzo

Corresponding authors: Flavia Guzzo and Stefano Negri (Department of Biotechnology, University of Verona, Strada Le Grazie 15, 37134, Verona, Italy). E-mails: [flavia.guzzo@univr.it](mailto:flavia.guzzo@univr.it); [stefano.negri@univr.it](mailto:stefano.negri@univr.it)

**Supplementary Table 2: Metabolites found in the serum of kiwifruit-treated mice. RT = retention time; *confirmed using authentic standards.**

|  | RT (min) | *m/z* (-) | Elemental formula | Putative identification | Fragments | Detected in fruit juice |
| --- | --- | --- | --- | --- | --- | --- |
| SERUM. C18 | 5.033 | 212.001 | C_8_H_7_NO_4_S | indoxyl sulfate |  | no |
|  | 5.450 | 896.905 |  |  |  | no |
|  | 0.814 | 191.055 | C_7_H_12_O_6_ | quinic acid* |  | **yes** |
|  | 14.007 | 313.178 |  |  |  | no |
|  | 4.962 | 258.991 | C_9_H_8_O_7_S | caffeic acid sulfate |  | no |
| SERUM. HILIC | 0.720 | 212.006 | C_8_H_7_NO_4_S | indoxyl sulfate |  | no |
|  | 0.615 | 172.994 | C_6_H_6_O_4_S | Hydroxybenzene sulfate |  | no |
|  | 1.115 | 258.995 | C_9_H_8_O_7_S | caffeic acid sulfate | 135.0469; 179.037 | no |
|  | 0.709 | 447.009 |  |  |  | no |
|  | 0.720 | 243.992 |  |  |  | no |
|  | 0.793 | 557.158 |  |  |  | no |
|  | 0.720 | 525.078 |  |  |  | no |
|  | 0.604 | 368.976 |  |  |  | no |
|  | 0.615 | 447.058 |  |  |  | no |
|  | 13.976 | 191.055 | C_7_H_12_O_6_ | quinic acid* |  | **yes** |
|  | 0.615 | 339.982 |  |  |  | no |
|  | 12.028 | 225.060 |  |  |  | no |
|  | 1.960 | 246.992 |  |  |  | no |
|  | 0.772 | 610.262 |  |  |  | no |
